# Exploring the role of CRISPR in advancing space biotechnology: challenges and solutions for human survival beyond earth

**DOI:** 10.3389/fbioe.2026.1887696

**Published:** 2026-08-26

**Authors:** Ubaid Ahmad

**Affiliations:** 1 Program in Biotechnology, Multidisciplinary and Interdisciplinary School, Chiang Mai University, Chiang Mai, Thailand; 2 Department of Chemistry, Faculty of Science, Chiang Mai University, Chiang Mai, Thailand

**Keywords:** closed-loop life-support systems, CRISPR-Cas9, human gene editing, microbial engineering, space agriculture, space biotechnology

## Abstract

As space exploration advances toward long-duration missions and the potential colonization of extraterrestrial environments, innovative strategies are required to address the physiological, agricultural, and environmental challenges associated with spaceflight. Clustered Regularly Interspaced Short Palindromic Repeats (CRISPR)-Cas9 gene-editing technology has emerged as a promising platform for overcoming these challenges through precise genetic modifications in humans, plants, and microorganisms. This review comprehensively examines the potential applications of CRISPR in space biotechnology, focusing on enhancing astronaut health by targeting genes associated with muscle atrophy, bone loss, radiation resistance, and immune function. It also discusses the potential of CRISPR to engineer crops capable of thriving under space conditions, including microgravity, elevated radiation, limited resources, and constrained lighting, through improved photosynthetic efficiency, nutrient utilization, and stress tolerance. Furthermore, the review explores the use of CRISPR-engineered microorganisms to support closed-loop life-support systems by improving air purification, water recycling, and waste biodegradation. Unlike previous reviews that primarily address individual aspects of CRISPR or space biotechnology, this review integrates advances in astronaut health, space agriculture, and microbial engineering within a unified framework while critically evaluating the associated ethical, technical, regulatory, and biosafety challenges. It also identifies current knowledge gaps and future research priorities to facilitate the responsible development and implementation of CRISPR-based technologies for long-duration space missions. Collectively, these insights highlight the transformative potential of CRISPR to support sustainable human exploration and long-term habitation beyond Earth.

## Graphical Abstract

**Figure:**
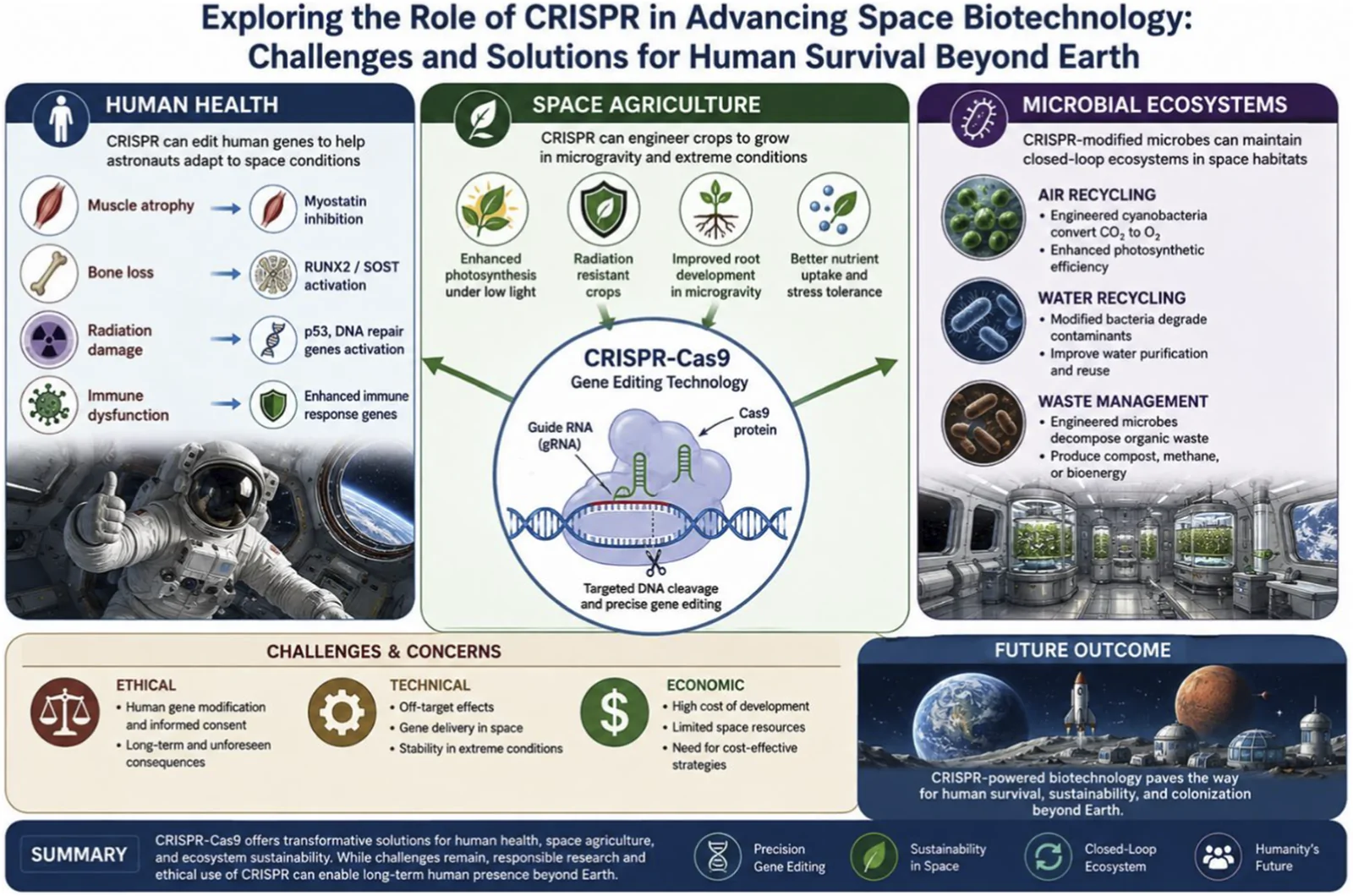

## 1 Introduction

Space exploration is one of humanity’s most ambitious endeavors, requiring major technological innovations to overcome the challenges associated with long-duration missions (Mammarella et al., 2025). As space missions move beyond Earth’s orbit and focus on Mars, the Moon, and farther destinations, astronauts face significant physical, psychological, and logistical challenges. Addressing these challenges is crucial for mission success, as it involves ensuring astronaut survival, health, and wellbeing, sustaining life in space, and managing resources effectively over extended periods.

### 1.1 Key challenges in long term space missions

Several fundamental challenges must be addressed for successful long-term space exploration:

1. Human health: One of the primary concerns for astronauts during missions beyond low Earth orbit is the impact of space conditions on their health (Hart, 2023). Microgravity causes bone demineralization and muscle atrophy, which can impair mobility and overall strength (Man et al., 2022). Furthermore, exposure to high-energy cosmic rays and solar radiation increases the risk of DNA damage, potentially contributing to a higher likelihood of cancer and genetic disorders (Cucinotta and Durante, 2006). The immune system also weakens in space, making astronauts more susceptible to infections and diseases (Sonnenfeld et al., 2003).
2. Food sustainability: Producing food in space presents a major logistical challenge. Spacecraft and habitats have limited space and resources, and traditional farming techniques are incompatible with space conditions like microgravity and limited natural resources (Carillo et al., 2020). Ensuring food security for astronauts on deep-space missions is critical to reducing dependency on resupply missions from Earth (Douglas et al., 2021).
3. Closed ecosystem management: Space habitats operate as closed-loop systems, meaning everything essential resources, including air, water, and food must be recycled or produced locally (Sabry, 2021). Managing these elements efficiently and sustainably is vital for the long-term success of space missions. Maintaining air quality, recycling water, and disposing of waste while keeping microbial contamination under control are complex tasks that require innovative solutions.

### 1.2 CRISPR technology as a game changer

**FIGURE 1:**
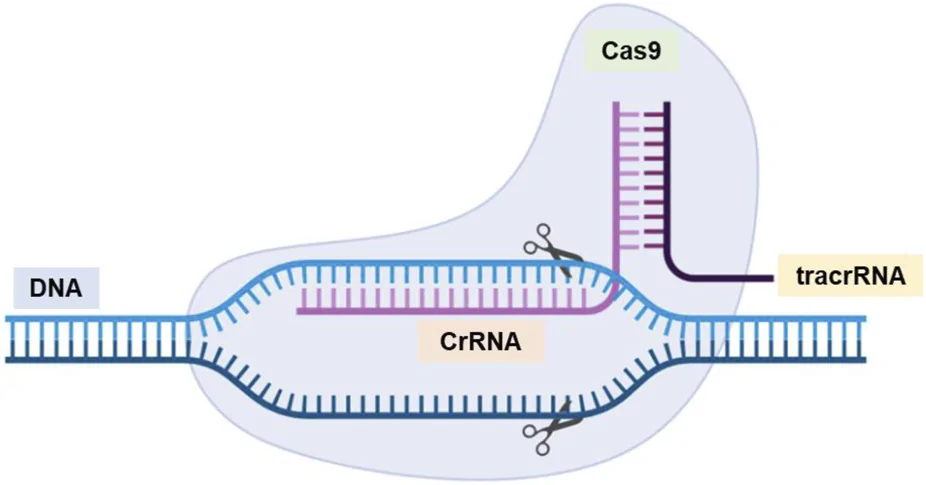
Schematic representation of the CRISPR-Cas9 gene-editing complex. The Cas9 protein (light blue) is guided by a dual-RNA structure composed of CRISPR RNA (CrRNA) and trans-activating CRISPR RNA (tracrRNA). The CrRNA directs the Cas9 complex to a complementary target sequence on the DNA (blue), allowing Cas9 to introduce a double-strand break at the specified location. The tracrRNA stabilizes the complex and facilitates CrRNA maturation (Navarro et al., 2025). The scissors icon indicates the DNA cleavage sites.

CRISPR-Cas9 (Clustered Regularly Interspaced Short Palindromic Repeats-associated protein 9) is a gene-editing technology that works by using a guide RNA (gRNA) to direct the Cas9 enzyme to a specific location within the DNA strand (Balasubramanian et al., 2024). The guide RNA is designed to match a complementary DNA sequence in the target gene (Rahman et al., 2024). Once the guide RNA binds to its target sequence on the DNA, Cas9 functions as molecular scissors, making a double-strand break at the exact location (Malla, 2025). After the DNA is cut, the cell’s natural repair mechanisms are triggered. Figure 1 illustrates schematic representation of the CRISPR-Cas9 gene-editing complex.

CRISPR-Cas9, a cutting-edge gene-editing technology (Redman et al., 2016), offers transformative potential for addressing many of these challenges in space exploration. By allowing precise alterations in the DNA of various organisms, CRISPR could revolutionize the ways we approach human health, space agriculture, and ecosystem management in space (Shubert, 2024).

1. Human health management: CRISPR can be used to modify human genes to help astronauts better adapt to the harsh conditions of space (Gouw and Szocik, 2020). Genetic modifications could potentially mitigate bone density loss, muscle atrophy, and enhance DNA repair mechanisms (Altieri et al., 2008). Additionally, CRISPR could strengthen the immune system, making astronauts more resilient to infections in space (Gouw and Szocik, 2020).
2. Agriculture in space: Through genetic modification of crops, CRISPR can create plants that are more resistant to space radiation, improve nutrient utilization efficiency, and maintain growth under low-light and microgravity conditions (Maffei et al., 2024). This would provide a reliable, sustainable food source for astronauts, reducing the need for frequent resupply missions from Earth.
3. Ecosystem management: CRISPR could also be used to engineer microorganisms that optimize life-support systems within space habitats (Mazhar et al., 2025). For instance, modified bacteria could improve air and water recycling processes, such as converting carbon dioxide into oxygen or efficiently breaking down waste (Abatenh et al., 2018). These innovations would help maintain a balanced, self-sustaining ecosystem aboard spacecraft and habitats.

### 1.3 Relevance: why CRISPR holds the potential for space biotechnology

**FIGURE 2:**
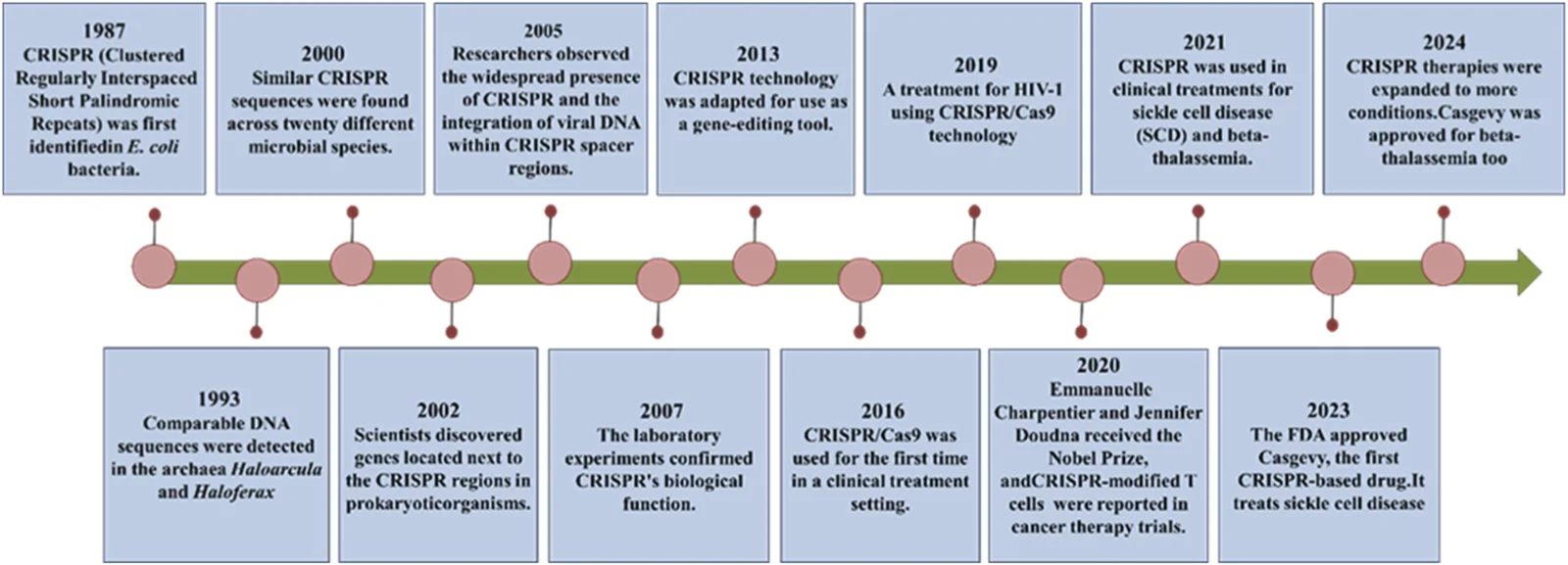
Timeline of major milestones in CRISPR discovery, development, and clinical applications.

CRISPR is an ideal tool for space biotechnology due to its precision, versatility, and relatively low cost (Safari et al., 2017). Unlike older genetic modification techniques, CRISPR enables targeted, precise changes to an organism’s DNA (Lino et al., 2018), which may help overcomethe unique challenges of space. Figure 2 shows the timeline of major milestones in CRISPR discovery, development, and clinical applications. The technology can be applied to humans, plants, and microbes alike, making it an adaptable solution for a wide range of space exploration needs (Onofri et al., 2025; Cui et al., 2024). With ongoing advancements in gene delivery systems and efficiency, CRISPR technology is becoming increasingly feasible for potential applications in future space mission (Lino et al., 2018).

Recent advances in CRISPR technology have substantially improved genome-editing precision, delivery efficiency, and safety through the development of high-fidelity Cas variants, optimized guide RNA design, and non-viral delivery platforms (Villiger et al., 2024; Pacesa et al., 2024; Wu et al., 2026). At the same time, emerging research in synthetic biology, regenerative life-support systems, and space biology has expanded the potential applications of genome editing for astronaut health, space agriculture, and engineered microbial ecosystems under spaceflight conditions (Maffei et al., 2024; Marshall et al., 2025; Stein, 2013). Despite these advances, most published studies focus on individual applications rather than providing an integrated assessment of CRISPR across the major pillars of space biotechnology.

### 1.4 Purpose of the paper

Recent advances in CRISPR technology and space biotechnology have highlighted the potential of gene editing to address challenges associated with long-duration space missions. While previous studies have explored the applications of CRISPR in human health, plant biotechnology, microbial engineering, or space biology individually, an integrated review that connects these diverse applications within the context of sustainable human space exploration remains limited. Moreover, ethical considerations, biosafety concerns, regulatory challenges, and future research priorities have often been discussed separately rather than within a unified framework.

Therefore, this review provides an integrated overview of the potential applications of CRISPR in space biotechnology. Specifically, it examines how CRISPR can be used to enhance astronaut health, engineer crops for extraterrestrial environments, and develop genetically modified microorganisms to support closed-loop life-support systems. In addition, this review critically discusses the ethical, technical, biosafety, and regulatory challenges associated with implementing CRISPR technologies during long-duration space missions, while identifying current knowledge gaps and future research directions. By integrating these multidisciplinary perspectives, this work establishes a framework to support the responsible development of CRISPR-based technologies for future lunar, Martian, and deep-space exploration.

## 2 Problem: effects of space travel on human health

**FIGURE 3:**
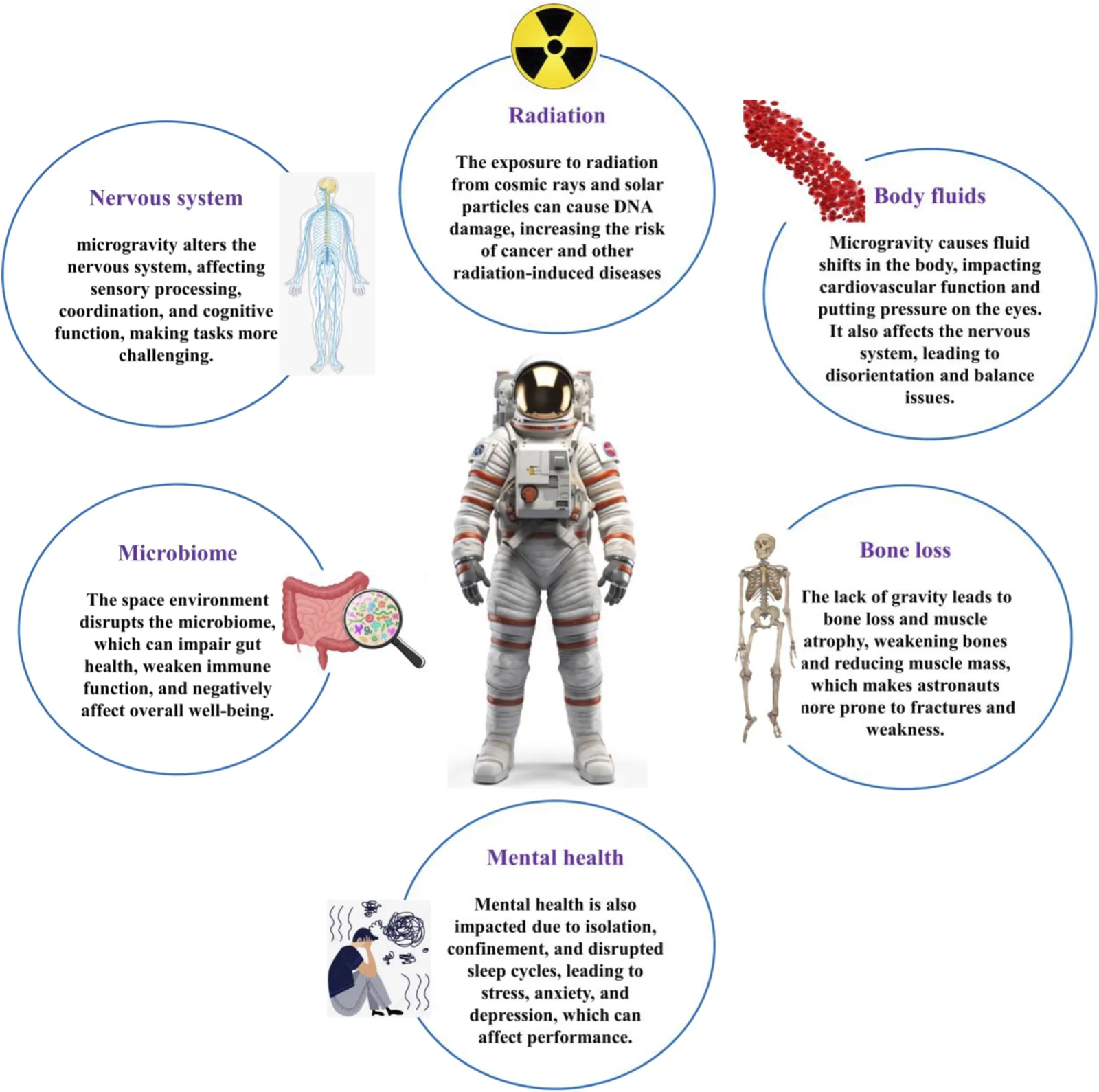
Summary of major physiological and psychological challenges encountered by astronauts during spaceflight.

Figure 3 summarizes of major physiological and psychological challenges encountered by astronauts during spaceflight. One of the most significant health issues faced by astronauts in space is the loss of muscle mass and bone density (Khan et al., 2021). In a microgravity environment, muscles and bones no longer experience the normal mechanical loading of Earth’s gravity, leading accelerated atrophy. This occurs because the mechanical load that usually stimulates muscle and bone growth is removed. As a result, astronauts experience severe muscle weakening, bone thinning, and a heightened risk of fractures, even after short missions (Sonnenfeld, 1998). This phenomenon is particularly problematic for missions lasting several months or years, such as those to Mars or deep-space missions.

Space travel has also been shown to weaken the immune system (Lv et al., 2023). Astronauts experience reduced immune function, which makes them more vulnerable to infections and diseases. One of the main causes of immune suppression in space is the lack of gravity, which affects the production and movement of immune cells throughout the body (Crucian et al., 2018). The stress and confined environment of space also play a role in reducing immune response, further exacerbating the risk of illness during long-term missions (Durante and Cucinotta, 2011).

Space travelers are exposed to high levels of radiation that do not exist on Earth due to the lack of a protective atmosphere and magnetic field (Mohan et al., 2022). Cosmic rays and solar radiation can damage cellular DNA, leading to mutations, cancer, and other radiation-induced health problems (Sathiya and Ramachandran, 2023). The prolonged exposure to this radiation increases the risk of long-term health complications, making it a significant challenge for deep-space missions that last months or years (Uddin et al., 2020).

### 2.1 Solution: how CRISPR could modify human genes to mitigate these issues

CRISPR technology offers a powerful tool for editing human genes to potentially prevent, mitigate, or alleviate health challenges associated with space travel (Gouw and Szocik, 2020). By precisely altering specific genes, CRISPR has the potential to address the underlying genetic mechanisms responsible for muscle and bone loss, radiation sensitivity, and immune system degradation (Sharma et al., 2001).

One of the genes that CRISPR could target for muscle preservation is myostatin. Myostatin is a negative regulator of muscle growth, meaning it limits muscle development (Li et al., 2020). By using CRISPR to deactivate the myostatin gene (Kamiya, 2012), astronauts could potentially maintain or even increase muscle mass during extended space missions. This type of gene editing could significantly reduce the muscle atrophy astronauts experience in microgravity.

Another key target for CRISPR is bone regeneration-related genes, such as RUNX2 or SOST, which play crucial roles in maintaining bone density (Blaber et al., 2013). By modifying these genes, CRISPR could help promote bone growth and prevent bone demineralization in space. For instance, enhancing the expression of genes related to bone formation and inhibiting those that cause bone loss could help astronauts maintain healthy bone structures during prolonged periods in space (Moreno-Villanueva et al., 2017).

One of the most significant risks of space travel is the exposure to radiation, which can damage DNA and increase the likelihood of genetic mutations (Okazaki, 2022). CRISPR could be used to enhance the body’s DNA repair mechanisms. For example, genes such as p53 which contributes to DNA damage response, and telomerase, which helps maintain chromosome stability, could theoretically be modified to improve cellular resistance to radiation-induced damage (Vodicka et al., 2021; Vukmirovic et al., 2020). CRISPR could also be used to boost the production of antioxidant enzymes that help protect cells from radiation-induced oxidative stress (Cervantes and Hong, 2016).

By modifying these pathways, astronauts could potentially improve their ability to respond to DNA damage caused by space radiation, thereby reducing the risk of radiation-associated health complications.

Space travel can cause immune system dysregulation, making astronauts more vulnerable to infections (Zhang and Cao, 2019). CRISPR could be employed to enhance immune responses by editing specific genes involved in immune cell function, such as those related to T cell production, antibody production, and immune memory. For example, CRISPR could be used to modify genes that control the activation of interleukins (important immune system signaling proteins) to improve immune responses to infections (Irvine et al., 2022).

Additionally, CRISPR could be used to enhance the expression of certain cytokines (molecules that promote immune cell communication) to help maintain immune system function in space (Nelson et al., 2010). This gene editing could ensure that astronauts' immune systems remain robust and capable of responding to pathogens during long missions.

Although these proposed strategies are supported by encouraging findings from terrestrial studies and preclinical gene-editing research, their translation to space medicine remains uncertain. Most current evidence has been generated under laboratory or simulated microgravity conditions, which cannot fully reproduce the combined effects of prolonged microgravity, cosmic radiation, psychological stress, and confined living environments experienced during long-duration space missions. Consequently, further validation under authentic spaceflight conditions is required before CRISPR-based genetic interventions can be considered for clinical implementation in astronauts.

## 3 Problem: challenges in growing crops in space

Space missions are constrained by the limited availability of resources, including water, nutrients, and space (Douglas et al., 2021). Traditional agricultural systems require large amounts of land, water, and other resources, which are difficult to provide in a confined space like a spacecraft or space habitat (Kumar et al., 2020). The need for resource-efficient farming systems is critical to ensuring the sustainability of long-duration missions. Space crops must thrive with minimal resources, and the use of genetically modified plants may be essential to achieving this goal (Ferl et al., 2002).

One of the most significant challenges for growing plants in space is the lack of gravity (Singh et al., 2017). Plants rely on gravity to orient their growth, guiding roots downward and shoots upward (a phenomenon known as gravitropism) (Kordyum et al., 2019). In microgravity, this mechanism is disrupted, leading to abnormal plant growth patterns. This can affect root development, nutrient uptake, and overall plant health. While microgravity does not completely prevent plant growth, it complicates the process, making it more difficult for plants to thrive (De et al., 2021).

In addition to low gravity, space crops must endure several types of environmental stress. These include exposure to higher levels of radiation, limited light conditions (due to the absence of a natural day-night cycle), and fluctuating temperatures. Radiation in space can damage plant cells, affecting growth and reducing the nutritional quality of crops (Wheeler et al., 2001). Moreover, the absence of natural sunlight and the constant conditions inside space habitats may limit photosynthesis and hinder crop development (Burgess et al., 2024). Nutrient limitations due to constrained resources and the lack of natural soil are also significant challenges for space agriculture (Khan et al., 2024).

### 3.1 Solution: how CRISPR can help in engineering crops for space environments

Space habitats often use artificial lighting, which may not be as effective as natural sunlight. CRISPR can be used to modify photosynthetic pathways to make plants more efficient in utilizing available light. By targeting genes involved in the light-harvesting complexes or the carbon fixation process, CRISPR could increase the plant’s efficiency in converting light into energy, even under low-light conditions (Badri et al., 2015). Additionally, CRISPR could modify plants to be more resilient to fluctuating light levels and extended periods of darkness, mimicking the conditions of space environments (Mazhar et al., 2025).

CRISPR can be used to enhance the expression of radiation-resistant genes in plants, such as those that help in repairing DNA damage caused by radiation (Farooq et al., 2024). For example, modifying plants to express more robust DNA repair enzymes or enhancing their antioxidant defense mechanisms could help them survive in the high-radiation environment of space.

Plants in space often experience stunted or abnormal growth due to the lack of gravity. CRISPR could be used to modify growth-regulating genes, such as those involved in gravitropism, to help plants better regulate their growth in microgravity (Cosgrove, 2024). Additionally, CRISPR could alter genes involved in root development, ensuring that plants can maintain healthy root systems even in the absence of gravity. Enhancing cell wall structure and water retention in plants may also help them adapt to the challenges of space farming (Yadav et al., 2023).

CRISPR can be applied to enhance the root architecture of plants, improving their ability to absorb nutrients from limited resources. For example, genes responsible for nitrogen fixation in plants can be enhanced to make crops more efficient at utilizing nitrogen, a crucial nutrient. Recent advances in plant genome editing have demonstrated the feasibility of engineering crops with enhanced tolerance to drought, salinity, and environmental stress under terrestrial conditions (Zafar et al., 2020; Everts et al., 2026). However, relatively few studies have evaluated CRISPR-engineered plants under authentic spaceflight conditions or prolonged microgravity exposure (Solomos, 1987). Consequently, the stability of these genetic modifications, their long-term productivity, and their nutritional performance in extraterrestrial environments remain important research priorities.

## 4 Problem: maintaining a sustainable and closed ecosystem in space

In a confined space environment, maintaining a constant supply of breathable air is vital. On Earth, plants and trees help maintain this balance by absorbing CO_2_ and releasing O_2_ (Fahrion et al., 2021). However, in space habitats, it is not feasible to rely on traditional plants due to space constraints and limited resources. Space habitats must rely on engineered microbial systems to perform essential functions such as CO_2_ absorption and oxygen production (Escobar and Nabity, 2017).

Without an efficient air recycling system, CO_2_ would accumulate in the closed habitat, which could lead to toxic conditions for astronauts (Maier and Gentry, 2015). Similarly, oxygen levels need to be maintained within a narrow range to support human life.

Water is another crucial resource for astronauts. Space habitats must recycle water used by astronauts for drinking, bathing, and other essential activities. Water purification and recycling systems must be highly efficient to minimize water scarcity in space. Microbial systems could play a key role in breaking down organic contaminants and maintaining a clean water supply (Masciandaro et al., 2013). However, these systems must be optimized to ensure that microbes effectively handle the breakdown of organic material without causing harmful build-up or contamination (Rai et al., 2022).

In addition to managing air and water, waste management is a critical component of space habitat sustainability. Astronauts generate a range of organic waste products, including food waste, human waste, and other biodegradable materials (Srivastava et al., 2020). Efficient decomposition of this waste is essential for maintaining habitat hygiene and preventing contamination of air and water. However, traditional waste management systems would require extensive space and resources, making them inefficient for long-term space missions. Microbial systems engineered for waste decomposition can provide a compact, sustainable solution to this problem (Haeder, 2022).

### 4.1 Solution: CRISPR-modified microbes for habitat sustainability

CRISPR can be used to genetically modify microbes that play essential roles in air, water, and waste management systems in space habitats. By optimizing microbial functions, CRISPR can enhance their ability to maintain a sustainable ecosystem in space environments. Below are some potential CRISPR-based solutions for improving microbial systems in space habitats:

Microbes like cyanobacteria and algae naturally absorb carbon dioxide and produce oxygen through photosynthesis (Koehle et al., 2023). CRISPR can be used to enhance these microbes’ photosynthetic efficiency, making them more efficient at recycling CO_2_ and generating oxygen in space habitats. By modifying genes responsible for photosynthetic pathways, CRISPR could increase the rate of CO_2_ fixation, helping maintain a stable oxygen-to-carbon dioxide ratio. Additionally, CRISPR could be used to optimize the microbes’ tolerance to space conditions, such as microgravity and radiation, ensuring their survival and efficient performance in space habitats (Vigil, 2003).

Microbes also play a critical role in water recycling systems by breaking down contaminants and maintaining water quality (Greenberger, 2024). Using CRISPR, scientists could engineer microbes that are highly efficient at degrading organic matter and purifying water. For instance, bacteria like Deinococcus *radiodurans*, known for its radiation resistance, could be genetically modified with CRISPR to enhance its capacity to survive in space and purify water used by astronauts (Singh et al., 2024). Similarly, microbes could be modified to improve nitrogen removal process, reducing the risk of nitrogen buildup in the habitat’s water system.

In space, astronauts generate organic waste that must be broken down and recycled (Srivastava et al., 2020). CRISPR can be used to genetically modify microbes such as bacteria, fungi, or yeast to optimize their ability to decompose human waste and food scraps. By targeting genes involved in lignin and cellulose breakdown, CRISPR can improve the efficiency of microbes in breaking down organic materials into simpler compounds, such as methane or compost, which can be used as fertilizer or recycled an energy source for other systems within the habitat (Amobonye et al., 2021). This reduces the amount of waste astronauts need to dispose of and contributes to the overall sustainability of the space habitat.

Microbes can also be modified to enhance their biodegradation capabilities, allowing them to break down a wider range of materials commonly found in space waste, including plastics and synthetic polymers (Santomartino et al., 2023). This could significantly reduce the environmental impact of waste in the habitat, ensuring a cleaner, safer environment for astronauts.

Although engineered microorganisms have demonstrated considerable promise for carbon recycling, water purification, and waste biodegradation in laboratory studies, their long-term behavior within closed extraterrestrial ecosystems remains largely unknown (Vigil, 2003; Howells et al., 2025; Everroad et al., 2023). Potential ecological interactions, horizontal gene transfer, microbial evolution, and system stability during extended missions require careful investigation before genetically modified microbial communities can be safely deployed in operational space habitats (Vigil, 2003; Correll and Worden, 2025). As shown in Table 1, CRISPR technology has diverse applications in space biology, including astronaut health, space agriculture, life-support systems, radiation protection, microbial biomanufacturing, and future space colonization.

**TABLE 1:** CRISPR-based applications in space exploration, including astronaut health, space agriculture, life-support systems, radiation protection, microbial biomanufacturing, and future colonization.

Application area	CRISPR target	Potential benefits	Major challenges	Current research status
Astronaut health	Myostatin, RUNX2, SOST, p53 (Lee et al., 2020)	Muscle preservation, bone protection, radiation resistance	Ethical concerns, off-target effects, delivery limitations	Mostly laboratory and preclinical studies
Space agriculture	Photosynthesis, gravitropism, stress-response genes	Improved crop growth, nutrient use, radiation tolerance (Nie et al., 2025)	Limited validation under real spaceflight	Experimental and simulated microgravity studies
Closed-loop life-support	Cyanobacteria, algae, Deinococcus, engineered microbes (Ellena et al., 2024)	Oxygen production, water purification, waste recycling	Genetic stability, containment, horizontal gene transfer	Early-stage experimental research
Radiation protection	DNA repair pathways, antioxidant genes	Enhanced DNA repair and reduced oxidative damage (Stahl-Rommel et al., 2021)	Long-term safety unknown	Mainly laboratory studies
Microbial biomanufacturing	Metabolic engineering pathways (Me et al., 2015)	Sustainable production of food, biomaterials, pharmaceuticals	Biosafety and regulatory concerns	Emerging research
Future space colonization	Integrated CRISPR systems	Sustainable habitats and long-duration missions	Ethical, technical, regulatory challenges	Conceptual with limited in-space validation

## 5 Ethical and technical challenges of CRISPR in Space

CRISPR technology presents extraordinary potential to address many challenges in space exploration, from human health to habitat sustainability. However, as with any groundbreaking technology, CRISPR raises both ethical and technical concerns that must be carefully considered before it is widely implemented in space missions. This section discusses key challenges in both areas and offers potential solutions to ensure that CRISPR’s use in space is safe, ethical, and technically feasible.

### 5.1 Problem 1: ethical concerns about human genetic modification for space adaptation

One of the most significant ethical challenges of CRISPR in space exploration is the genetic modification of humans for space adaptation (Szocik et al., 2021). Long-duration space missions, such as those to Mars, may expose astronauts to extreme environmental stressors like microgravity, radiation, and limited resources. Genetic modifications aimed at adapting humans to these harsh conditions such as enhancing bone density, muscle mass, or radiation resistance could be highly beneficial, but they raise significant ethical questions (Munsie and Gyngell, 2018).

Modifying human genes for space adaptation, especially if it involves somatic changes (i.e., changes made to the individual rather than their offspring), raises questions about the morality of altering the human genome, particularly when the long-term consequences of such modifications are not fully understood (National Academies of Sciences et al., 2017).

There are concerns about whether astronauts can provide truly informed consent to undergo genetic modification for space missions, especially if the risks and benefits are not fully understood (Szocik et al., 2020). The ethical implications of making irreversible genetic changes for the benefit of space exploration, particularly when astronauts may not be fully aware of the potential long-term consequences, need to be addressed.

### 5.2 Solution: ethical frameworks for gene editing

To address these concerns, a comprehensive ethical framework is needed to govern human genetic modifications in space.

Strict guidelines and informed consent processes must be developed to ensure that astronauts fully understand the potential risks and benefits of genetic modifications. This should include clear communication about the purpose of gene editing, possible side effects, and long-term health consequences, as well as the option to opt out of such procedures if desired (Coller, 2019; Conley et al., 2025).

Ethical oversight should be put in place to regulate and monitor somatic gene editing. International space agencies, such as NASA, and space exploration companies should collaborate with bioethicists, scientists, and policymakers to develop protocols for genetic editing that prioritize astronaut safety while minimizing the risks of unintended consequences (Jasanoff et al., 2025; Baylis, 2025; Freedman and Tsai, 2026).

Ethical guidelines should also include mechanisms for minimizing potential health risks associated with genetic modifications, such as regular monitoring of astronauts’ health during and after space missions. Furthermore, genetic modifications should be reversible or should not carry irreversible risks to astronauts' long-term health or fertility (Gibelli et al., 2025; Cole et al., 2025; Van den Nieuwenhof et al., 2024).

Beyond ethical oversight, international regulatory frameworks and planetary protection policies should guide the responsible application of CRISPR technologies in space missions. Existing international agreements, including the Outer Space Treaty, emphasize the peaceful exploration of outer space and the responsibility of nations to avoid harmful contamination of celestial bodies. Likewise, the Committee on Space Research (COSPAR) provides planetary protection guidelines aimed at preventing forward contamination of extraterrestrial environments and backward contamination of Earth. Future applications of CRISPR-based gene editing in astronauts, plants, and microorganisms should therefore comply with international biosecurity standards, planetary protection principles, and transparent governance frameworks. International collaboration among space agencies, regulatory authorities, scientists, and bioethicists will be essential to establish harmonized policies that ensure both scientific progress and the safe, responsible use of genome-editing technologies during long-duration space exploration.

### 5.3 Problem 2: technical limitations of CRISPR for space applications

Despite its transformative potential, several technical limitations must be addressed before CRISPR can be safely and reliably implemented in space biotechnology. Although CRISPR-Cas systems have demonstrated remarkable precision and efficiency under laboratory conditions, their performance in the space environment remains insufficiently understood. Factors such as microgravity, cosmic radiation, prolonged mission duration, and limited onboard medical and laboratory resources may influence genome-editing efficiency, accuracy, and long-term stability. Addressing these challenges is essential to ensure the safe application of CRISPR technologies during future lunar, Martian, and deep-space missions (Villiger et al., 2024; Pacesa et al., 2024; Wadhwa et al., 2024; Kim et al., 2019).

One of the primary technical concerns is the occurrence of off-target mutations, in which the CRISPR-Cas system introduces unintended genetic modifications at genomic sites that share partial sequence similarity with the intended target. Such unintended edits may disrupt essential genes or regulatory elements, potentially leading to impaired cellular function, genomic instability, or unforeseen biological consequences. Although advances in guide RNA design, computational prediction tools, and high-fidelity Cas variants have substantially improved editing specificity, eliminating off-target effects remains an important challenge, particularly for applications involving human genome editing (Villiger et al., 2024; Pacesa et al., 2024; Wilbie et al., 2019; Yin et al., 2017).

Another major limitation is the efficient delivery of CRISPR components into target cells and tissues. Successful genome editing depends on the reliable delivery of Cas proteins, messenger RNA, or gene-editing constructs using viral vectors, lipid nanoparticles, or other delivery platforms (Kaupbayeva et al., 2024; Ma et al., 2026; Uzakova et al., 2025). However, each delivery strategy has inherent limitations related to delivery efficiency, payload capacity, immunogenicity, and long-term safety (Aksoyalp et al., 2024; López Garzón et al., 2025). These challenges may be further exacerbated under spaceflight conditions, where microgravity and radiation can alter cellular physiology, immune responses, and the performance of delivery systems (Madigan et al., 2023; Wang et al., 2025).

The long-term stability and safety of edited genomes also remain significant concerns. Continuous exposure to ionizing radiation and other environmental stressors during prolonged space missions may influence DNA repair pathways, increase genomic instability, or alter the persistence of intended genetic modifications. Consequently, the durability and safety of CRISPR-mediated genetic changes require comprehensive long-term evaluation before their routine implementation in astronauts, crops, or engineered microorganisms (Villiger et al., 2024; Jung et al., 2024; Zheng et al., 2024; Kim et al., 2025).

Furthermore, genome-editing efficiency varies considerably among different organisms, tissues, and cell types. Factors such as chromatin accessibility, DNA repair mechanisms, target sequence characteristics, and cellular metabolic states can all influence editing outcomes. Achieving consistent, reproducible, and highly efficient genome editing therefore remains a major technical challenge for many space biotechnology applications (Villiger et al., 2024; Sreejalekshmi et al., 2025; Antony et al., 2025).

Finally, experimental validation of CRISPR technologies under authentic spaceflight conditions remains limited. Most available studies have been performed under terrestrial laboratory conditions or simulated microgravity environments, which cannot fully reproduce the combined effects of microgravity, cosmic radiation, confinement, and long-duration missions. Additional research conducted aboard orbital platforms and future deep-space missions will therefore be essential to validate the safety, efficiency, and long-term reliability of CRISPR-based interventions in realistic space environments (Onofri et al., 2025; Stein, 2013; Macauley, 2005).

#### 5.3.1 Solution: strategies to improve the safety and reliability of CRISPR in space

Several technological advances are being developed to overcome these limitations and improve the safety of CRISPR-based applications for space exploration. The use of high-fidelity Cas nucleases and optimized guide RNA design has significantly reduced off-target genome editing, thereby improving editing precision. Advances in non-viral delivery systems, particularly lipid nanoparticles and biodegradable nanocarriers, offer safer and more efficient alternatives for delivering CRISPR components while reducing immunogenicity and minimizing the risks associated with viral vectors. Comprehensive pre-flight validation using simulated microgravity platforms, radiation exposure models, and relevant biological systems should also be performed to evaluate genome-editing performance under space-like conditions before clinical or operational deployment. In addition, long-term genomic monitoring of genetically modified humans, plants, and microorganisms will be essential to detect unintended genetic alterations, evaluate genomic stability, and ensure the sustained safety and effectiveness of CRISPR-based interventions during extended space missions. Continued advances in genome-editing technologies, delivery platforms, and safety assessment frameworks will be critical for translating CRISPR from an experimental research tool into a reliable biotechnology platform capable of supporting sustainable human exploration beyond Earth (Onofri et al., 2025; Villiger et al., 2024; Pacesa et al., 2024; Jung et al., 2024; Zheng et al., 2024). Beyond supporting human space exploration, these technological advances may also accelerate innovations across terrestrial biotechnology. Improvements in genome-editing precision, autonomous molecular diagnostics, synthetic biology, regenerative medicine, and closed-loop biomanufacturing developed for space applications may ultimately benefit healthcare, sustainable agriculture, environmental remediation, and industrial biotechnology on Earth. Consequently, investment in space biotechnology has the potential to generate scientific and technological advances with far-reaching societal benefits beyond extraterrestrial exploration (Barrangou et al., 2016; Piergentili et al., 2021).

### 5.4 Problem 3: resource and cost constraints for space based CRISPR applications

Space missions have limited resources and high costs associated with sending equipment and personnel into space (Nickerson et al., 2024). These constraints pose a major challenge for the application of CRISPR in space biotechnology, as it may be difficult to justify the investment in gene-editing technologies when resources are already scarce.

Space habitats are constrained by factors such as power, storage, and equipment, meaning that CRISPR technologies need to be compact, efficient, and sustainable. Any space-based application of CRISPR must be designed to fit within the tight space and resource limitations of space missions.

The development, manufacturing, and deployment of CRISPR-based tools and technologies are expensive (Cheng et al., 2025). For long-duration space missions or colonization efforts, the financial cost of developing and deploying CRISPR technology in space must be minimized to ensure its sustainability.

#### 5.4.1 Solution: cost effective CRISPR strategies

One potential solution to reduce costs is the use of automated systems to perform CRISPR gene editing (Cheng et al., 2025). These systems could be developed to work autonomously, reducing the need for astronaut intervention and the associated labor costs. This would also ensure that gene editing can be performed efficiently, without requiring significant human input.

Researchers are working on more efficient and cost-effective gene-editing tools. For example, tools that require fewer components or less energy to work could be more suitable for space missions. By optimizing CRISPR technology for space use, scientists can reduce both the cost and the complexity of gene editing in space (Doudna and Charpentier, 2014).

Another strategy is to make use of existing infrastructure in space. For example, space agencies could repurpose current equipment used for other life-support functions (e.g., water recycling or waste management) to also support CRISPR gene editing. This would reduce the need for additional specialized equipment and minimize the overall cost.

### 5.5 Limitations of the present review

Although this review provides a comprehensive overview of CRISPR applications in space biotechnology, several limitations should be acknowledged. First, much of the available evidence is derived from terrestrial laboratory experiments or simulated microgravity models, whereas relatively few studies have evaluated CRISPR technologies under authentic spaceflight conditions (Musunuru et al., 2021; Tomsia et al., 2024). Second, because space biotechnology remains an emerging field, many proposed applications—including human genome editing for astronaut adaptation and genetically engineered life-support systems—remain largely conceptual and have not yet undergone long-term experimental validation (Onofri et al., 2025; Fatehi et al., 2023). Third, considerable variability exists among published studies regarding CRISPR platforms, experimental models, delivery strategies, and biological endpoints, making direct comparisons difficult (Afshinnekoo et al., 2020; Rutter et al., 2020). Finally, many recent technological developments have yet to be translated into operational space missions, limiting the availability of real-world performance data. Future research should therefore prioritize standardized experimental protocols, long-duration spaceflight validation, multidisciplinary collaboration, and comprehensive safety assessments to facilitate the responsible implementation of CRISPR technologies in future lunar, Martian, and deep-space missions.

## 6 Future directions and research needs

While CRISPR shows great promise, there are still unanswered questions that need to be addressed in the context of space exploration:

1. Long-term effects of CRISPR in space: The long-term effects of gene editing on both humans and microorganisms in space are still not well understood. Research should focus on how genetic modifications hold up over extended periods in space, especially under the harsh environmental conditions of space.
2. Full genetic edits for space adaptation: While we have started modifying genes related to bone density, muscle maintenance, and immune function, a more comprehensive approach to space adaptation may require full genetic edits (Villiger et al., 2024). What are the necessary genes for optimizing human survival on Mars or beyond? Identifying these genes and testing their functionality is a major area of interest.
3. Validation under authentic spaceflight conditions: Most CRISPR studies have been conducted under terrestrial laboratory or simulated microgravity conditions. Future investigations should evaluate genome-editing efficiency, precision, safety, and long-term stability aboard orbital platforms and future lunar or Martian missions.
4. Development of advanced delivery systems: Continued research is needed to optimize non-viral delivery platforms, including lipid nanoparticles and biodegradable nanocarriers, to improve delivery efficiency while minimizing immunogenicity and off-target effects under spaceflight conditions.
5. Ethical and regulatory frameworks: International collaboration among space agencies, regulatory authorities, bioethicists, and scientists will be essential to establish standardized ethical guidelines, biosafety regulations, and governance frameworks for the responsible use of genome editing during long-duration space missions.

## 7 Conclusion

In conclusion, CRISPR technology offers significant potential in addressing some of the most critical challenges facing space exploration. As humanity ventures into long-term space missions, including lunar bases and Mars exploration, CRISPR-based approaches may contribute to maintaining human health, improving food production, and supporting habitat sustainability under extreme space conditions.

CRISPR-mediated modification of genes associated with bone density, muscle maintenance, and immune function holds promise for mitigating some adverse effects of space travel, including muscle atrophy, bone loss, and immune dysfunction.

Through targeted genetic approaches, CRISPR could potentially help astronauts better adapt to the challenges of microgravity and space radiation, although extensive validation is required before such applications can be considered for human spaceflight.

CRISPR-based crop engineering may support sustainable space agriculture by enabling plants to tolerate environmental stresses such as low gravity, limited light availability, radiation exposure, and nutrient constraints. By improving photosynthetic efficiency, stress resistance, and nutrient utilization, CRISPR-edited plants could contribute to reliable food production systems for long-duration missions while reducing dependence on Earth-based resupply.

CRISPR-modified microorganisms could contribute to maintaining closed-loop life-support systems within space habitats. By improving microbial functions involved in air recycling, water purification, and waste degradation, engineered microorganisms may support the development of more sustainable and self-sufficient space ecosystems. These innovations would be crucial for missions to distant planets, such as Mars or the Moon.

The potential impact of CRISPR technology in space biotechnology extends beyond extraterrestrial applications. By enabling genetic modifications that address the physiological, agricultural, and environmental challenges of space, CRISPR represents a critical tool for ensuring the success of long-term space missions and human adaptation to space environments.

Importantly, the implications of CRISPR in space biotechnology extend beyond extraterrestrial applications. Advances driven by space-focused research may accelerate innovations in terrestrial biotechnology, including regenerative medicine, synthetic biology, environmental remediation, and sustainable agriculture, thereby generating broad societal benefits beyond space exploration.

Despite its promising potential, significant ethical, technical, regulatory, and financial challenges must be addressed before CRISPR can be implemented in space environments. Precision in gene editing, safe delivery mechanisms, and ethical frameworks for human genetic modification will be essential in ensuring that CRISPR is used responsibly and effectively.

As space exploration continues to progress, CRISPR, together with complementary technologies such as synthetic biology and advanced biomanufacturing, may become an important component of future strategies aimed at supporting long-term human presence beyond Earth. The continued development of CRISPR, along with complementary technologies such as synthetic biology and gene drives, will likely pave the way for the colonization of other planets, making long-term human presence in space a reality.
